# Use of machine learning in osteoarthritis research: a systematic literature review

**DOI:** 10.1136/rmdopen-2021-001998

**Published:** 2022-03-16

**Authors:** Marie Binvignat, Valentina Pedoia, Atul J Butte, Karine Louati, David Klatzmann, Francis Berenbaum, Encarnita Mariotti-Ferrandiz, Jérémie Sellam

**Affiliations:** 1 Department of Rheumatology, Hôpital Saint-Antoine, Assistance Publique – Hôpitaux de Paris (AP-HP), Centre de Recherche Saint-Antoine, Inserm UMRS_938, Assistance Publique – Hôpitaux de Paris (AP-HP), Sorbonne Universite, Paris, France; 2 Bakar Computational Health Science Institute, University of California, San Francisco, California, USA; 3 Immunology Immunopathology Immunotherapy UMRS_959, Sorbonne Universite, Paris, France; 4 Center for Intelligent Imaging (CI2), Department of Radiology and Biomedical Imaging, University of California, San Francisco, California, USA; 5 Biotherapy (CIC-BTi) and Inflammation Immunopathology-Biotherapy Department (i2B), Hôpital Pitié-Salpêtrière, AP-HP, Paris, France

**Keywords:** Artificial Intelligence, Machine Learning, Osteoarthritis, Systemic Literature Review

## Abstract

**Objective:**

The aim of this systematic literature review was to provide a comprehensive and exhaustive overview of the use of machine learning (ML) in the clinical care of osteoarthritis (OA).

**Methods:**

A systematic literature review was performed in July 2021 using MEDLINE PubMed with key words and MeSH terms. For each selected article, the number of patients, ML algorithms used, type of data analysed, validation methods and data availability were collected.

**Results:**

From 1148 screened articles, 46 were selected and analysed; most were published after 2017. Twelve articles were related to diagnosis, 7 to prediction, 4 to phenotyping, 12 to severity and 11 to progression. The number of patients included ranged from 18 to 5749. Overall, 35% of the articles described the use of deep learning And 74% imaging analyses. A total of 85% of the articles involved knee OA and 15% hip OA. No study investigated hand OA. Most of the studies involved the same cohort, with data from the OA initiative described in 46% of the articles and the MOST and Cohort Hip and Cohort Knee cohorts in 11% and 7%. Data and source codes were described as publicly available respectively in 54% and 22% of the articles. External validation was provided in only 7% of the articles.

**Conclusion:**

This review proposes an up-to-date overview of ML approaches used in clinical OA research and will help to enhance its application in this field.

**Key messages**

**What is already known about this subject?**

- This is the first systemic literature review of machine learning and osteoarthritis.

**What does this study add?**

- Most (85%) of the machine learning articles focused on knee osteoarthritis, and radiological data investigation predominated clearly over clinical or biological data.
- Almost half of the selected articles described use of the osteoarthritis initiative database, and external validation was poorly used (7% of the articles).

**How might this impact on clinical practice or further developments?**

- Application of machine learning is needed in other sites of osteoarthritis such as the hand or foot osteoarthritis, and new cohorts need to be established.
- Improving reproducibility and understanding of machine learning in the osteoarthritis field is needed.

## Introduction

The development of artificial intelligence (AI), especially machine learning (ML), in healthcare has led to important improvements and discoveries, notably in rheumatology and osteoarthritis (OA).^1–3^ There are many definitions for AI, but it could be summarised as the ability for a computer system to perform intellectual tasks normally requiring human skills.

**Figure 1:**
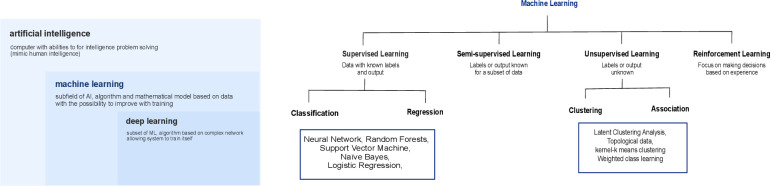
Definition of artificial intelligence (AI), machine learning (ML)and deep learning and summary of the different algorithms used in ML.

AI includes ML^4^ ^5^ defined as the ability to ‘learn’ or progressively improving performance from data. ML methods can be supervised or unsupervised. In supervised analysis, outcomes are known, and data are labelled. Conversely, in unsupervised ML, the outcomes and data are unknown and unlabeled. Two additional categories have been further proposed: semisupervised learning and reinforcement learning, with the outcome only partially known.^6^ Semisupervised learning models consist of a mix of labelled and unlabeled data and are based on weak supervision, with limited labelled data used to provide information and supervision for unlabeled data. However, reinforcement learning is an ML paradigm in which learning occurs iteratively via a series of trial-and-error cycles to maximise the reward received after each trial and therefore improve the learning. Supervised ML methods are the most commonly used in medicine and healthcare.^7^ Among supervised ML, we can distinguish different algorithms such as random forest, support vector machine, and convolutional neural networks according to the type of analyses^8^ (figure 1). Deep learning (DL) is a subtype of ML based on multiple layers of a neuron-architecture network allowing the model to improve and train itself and leading to high accuracy via high-level feature extraction from data.^9^

In these ML algorithms, we can distinguish explainable ML models (eg, linear models, naïve Bayes, logistic regression) from unexplainable ML models (eg, decision tree models, neural network, support vector machine), also known as interpretable ML or ‘white-box’ models for which the results of the algorithm can be understood by human intelligence. In contrast, unexplainable ML models (or ‘black-box models’) are algorithms for which, theoretically, one cannot possibly explain how and why the algorithm achieved a specific decision. Interpretability tools and methods developed to improve the models explainability include gradient-based methods for convolutional neural networks (eg, gradient class activation map^10^), shapley additive explanation for decision-tree models^11^ and local interpretable model-agnostic explanation.^12^ These interpretability tools are important because they help determine which features contribute to a specific model decision.^13^

In supervised ML, usually, the data used are separated in two parts with a training dataset to teach the algorithm and a testing dataset to test the performance of the model. Performance in ML is evaluated with different prediction metrics such as accuracy, sensitivity, specificity and precision. Finally, a step of validation is needed to assess the reproducibility of the dataset and avoid overfitting, which can be applied by k-fold cross-validation, bootstrap, leave one-out or splitting dataset, or using external data. Validation plays a key role in ML study because reproducibility remains one of the main critical issues and challenges in ML.^14^

In rheumatology, ML analyses have improved our knowledge of patient trajectories via disease and care modelling as well as response to treatment or disease phenotyping predictions with immunological signatures.^15–17^ With the growing number of studies using AI or ML in rheumatology,^18^ heterogeneous methodologies have been identified. Thus, the European League Against Rheumatism proposes ‘Points to Consider’ to improve the approach for better results.^19^

ML is also applied in the field of OA, especially with the establishment of large cohorts such as the OA Initiative (OAI),^20^ an observational cohort study of knee OA; the Multi-Centre Osteoarthritis Study (MOST),^21^ a longitudinal prospective and observational study of knee OA; and the Cohort Hip and Cohort Knee (CHECK),^22^ a prospective observational cohort of knee and hip OA. However, despite its trending and increasing applications, ML remains an emerging field with incredible potential but also limitations. Thus, a better delineation and understanding of the ML methods used in OA is needed.

The aim of this systemic literature review was to give a comprehensive overview of ML in clinical OA.

## Materials and methods

### Information sources and search strategy

The systemic literature review was conducted in accordance with the Preferred Reporting Items for Systematic Reviews and Meta-analyses guidelines^23 24^ and was registered in the International Prospective Register of Systemic Review PROSPERO^25^ (CRD42021272975). Articles in MEDLINE PubMed were searched beginning on 9 July 2021, by using the following MeSH and standard terms ((*human [MeSH Terms]) AND (osteoarthritis [MeSH Terms]) AND ((algorithms [MeSH Terms]) OR (machine learning) OR (“information systems”[MeSH Terms]) OR (“artificial intelligence”[MeSH Terms]) OR (artificial intelligence*)). The choice of these terms was motivated by the complexity of the definition of ML and by a willingness to be as exhaustive as possible. The definition of ML was based on classification and algorithms listed by scikit-machine learning module documentation.^26^

### Eligibility criteria

We searched for and included only original articles using AI and ML algorithms with clinical application in human OA. We excluded articles in a language other than English and articles related to surgery (especially those related to robotics and outcomes after total knee replacement); articles focused on locomotor metrics related to physical therapy outcomes; articles related to therapeutics, spine OA and temporo-mandibular OA; basic research articles and/or studies using murine models; basic cellular or molecular biology articles; and articles related to basic and fundamental imaging as well as theoretical ML (table 1).

**Table 1:** Inclusion and exclusion criteria of the systemic literature review

Inclusion criteria	OA Human Machine learning algorithms
Exclusion criteria	Review and meta-analysis Non-clinical OA articles Surgery. Non-applied radiology. Physical therapy. Treatments. Experimental OA Molecular biology. Murine model. Cell biology. Temporo-mandibular OA Spine OA Non-available articles Full text not available. Non-English articles.

OA, osteoarthritis.

### Article selection and data extraction

Two article selection steps based on eligibly criteria have been used. A first selection was based on abstracts and a second selection on full-text articles. The final choice of articles was independently validated by the three coauthors. Data have been extracted data by using a csv file extraction from the National Center for Biotechnology Information (NCBI) database.

For each article we collected the following:

- The domain of application of the article: diagnosis, prediction, phenotyping, severity, and progression of OA.
- Number of patients, year of publication, localisation of OA (knee, hand, foot and/or hip), main ML method of analysis and the notion of supervised and unsupervised analysis, use of DL, explainable ML and interpretability tools, type of data analysed (clinical, biological and imaging data), name of the cohort, presence of testing and training dataset, validation method when used and data and source code availability.

### Statistical analysis

Descriptive analyses, tabulation, visual display of the results and subgroup analysis were performed with R V.4.1.1 (2021-08-10). Graphical visualisation involved using the software BioRender and Affinity Designer.

## Results

### Selection flow chart

**Figure 2:**
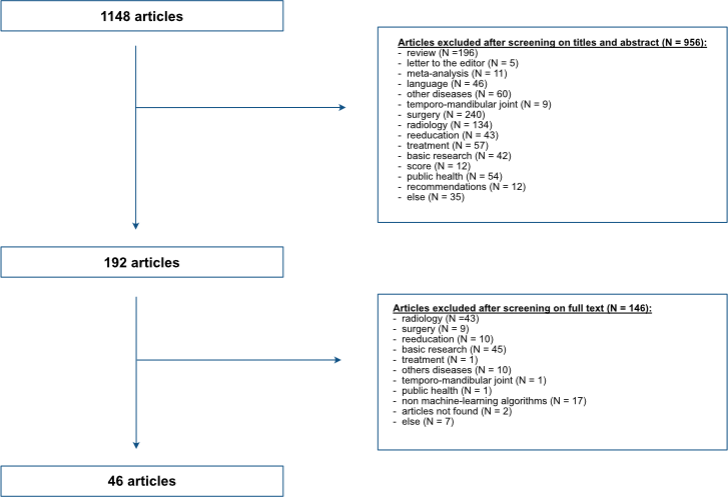
Flow of article selection.

We retrieved 1148 articles from the search. The flow chart is in figure 2. In the first selection step based on the abstracts for 1148 articles, 956 articles were excluded, including 196 reviews, 240 articles related to surgery, 134 on fundamental and theoretical imaging, 43 on reeducation, 57 related to treatment, 42 on basic research and 60 related to other diseases, and 192 articles remained. After reading complete articles, we excluded 43 articles related to theoretical radiology, 9 to surgery outcomes and robotics, 10 to reeducation outcomes, 45 to molecular biology and 10 to other diseases. We finally selected and analysed 46 articles^9 27–72^ (online supplemental table S1).

10.1136/rmdopen-2021-001998.supp1 Supplementary data

### Systematic literature overview

**Figure 3:**
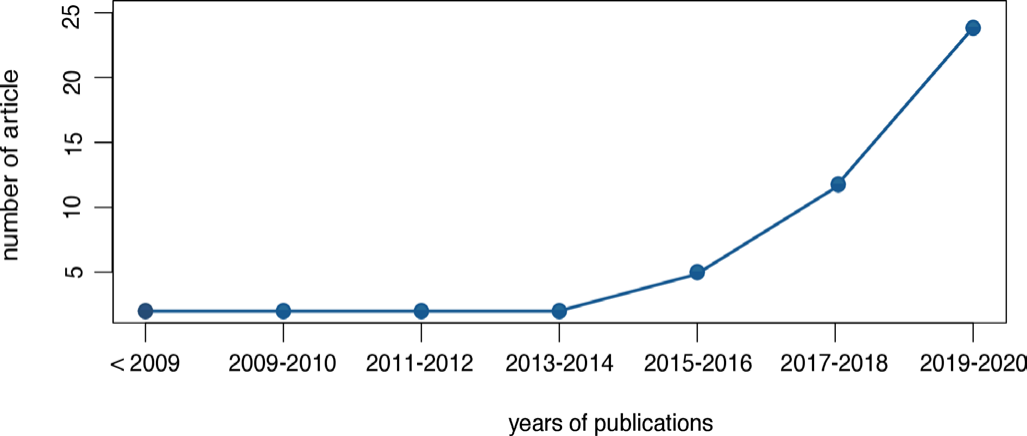
Evolution of publications related to machine learning and osteoarthritis.

Among the 46 identified articles published between 2007 and 2021, 74% were published after 2017 (figure 3); 12 were devoted to the diagnosis of OA at an early stage, 7 to the prediction of developing OA in healthy volunteers, 4 to the identification of OA phenotypes, 12 an automated estimation of OA structural severity classification and 11 to the identification of the progression of OA, notably patients with rapid disease progression. A complete descriptive analysis of the review is summarised in table 2 and online supplemental table S2.

**Table 2:** Descriptive analysis of 46 selected articles

	Overall	Diagnosis^27 30–38 40 70^	Prediction^39 41–45 71^	Phenotypes^29 46 47 69^	Severity^48–59^	Progression[28 60–68 72
	(N=46)	(N=12)	(N=7)	(N=4)	(N=12)	(N=11)
No of patients						
Mean	**1 359**	978	1 254	518	1 803	1 662
Median (range)	**525 (18–5 749**)	263 (60–5 749)	601 (68–4 796)	559 (102–852)	942 (18–4 504)	728 (100–4 796)
Year of publication						
Mean	**2017**	2017	2015	2018	2018	2018
Median (range)	**2019 (2007–2021**)	2018 (2012–2020)	2017 (2008–2020)	2018 (2015–2019)	2020 (2007–2021)	2019 (2012–2020)
Type of method						
Supervised	**40** (**87%**)	11 (92%)	7 (100%)	1 (25%)	11 (92%)	10 (91%)
Unsupervised	**4** (**9%**)	0 (0%)	0 (0%)	3 (75%)	0 (0%)	1 (9%)
Semi-supervised	**2** (**4%**)	1 (8%)	0 (0%)	0 (0%)	1 (8%)	0 (0%)
Method subtype						
Most frequently used	Convolutional neural network artificial neural network	Convolutional neural network random forest	Elastic net WND-CHARM	Latent class analysis topological data analysis	Convolutional neural network densely connected convolutional network	Logistic regression convolutional neural network
Mixed algorithms						
Yes	**13** (**28%**)	4 (33%)	0 (0%)	0 (0%)	4 (33%)	5 (45%)
No	**33** (**72%**)	8 (67%)	7 (100%)	4 (100%)	8 (67%)	6 (55%)
Deep learning						
Yes	**16** (**35%**)	4 (33%)	0 (0%)	0 (0%)	9 (75%)	3 (27%)
No	**30** (**65%**)	8 (67%)	7 (100%)	4 (100%)	3 (25%)	8 (73%)
Explainable model						
	**15** (**33%**)	3 (25%)	3 (43%)	4 (100%)	0 (0%)	5 (45%)
Unexplainable model						
	**31** (**67%**)	9 (75%)	4 (57%)	0 (0%)	12 (100%)	6 (55%)
Interpretability tools	**10** (**22%**)	2 (17%)	1 (14%)	0 (0%)	4 (33%)	3 (27%)
OA localisation						
Knee	**39** (**85%**)	10 (83%)	5 (71%)	3 (75%)	10 (83%)	12 (100%)
Hip	**7** (**15%**)	1 (8%)	2 (29%)	1 (25%)	2 (17%)	1 (9%)
Hand	**0** (0%)	0 (0%)	0 (0%)	0 (0%)	0 (0%)	0 (0%)
Foot	**0** (**0%**)	0 (0%)	0 (0%)	0 (0%)	0 (0%)	0 (0%)
Clinical data	**19** (**41%**)	2 (17%)	3 (43%)	4 (100%)	3 (25%)	7 (64%)
Biological data	**7** (**15%**)	4 (33%)	1 (14%)	1 (25%)	0 (0%)	1 (9%)
Serum	6 (13%)	3 (25%)	1 (14%)	1 (25%)	0 (0%)	1 (9%)
Synovium	2 (4%)	2 (16%)	0 (0%)	0 (0%)	0 (0%)	0 (0%)
Imaging data	**34** (**74%**)	6 (50%)	6 (86%)	3 (75%)	10 (83.3%)	9 (82%)
X-ray	28 (61%)	5 (42%)	5 (70%)	3 (75%)	9 (75%)	6 (55%)
MRI	10 (22%)	1 (8%)	2 (17%)	2 (50%)	1 (8%)	4 (36%)
Multiple data						
Yes	**11** (**24%**)	0 (0%)	2 (28.6%)	3 (75%)	1 (8.3%)	5 (45%)
No	**35** (**76%**)	12 (100%)	5 (71%)	1 (25%)	11 (91.7%)	6 (55%)
Training and testing sets						
Yes	**29** (**63%**)	9 (75%)	6 (86%)	0 (0%)	10 (83%)	4 (36%)
No	**17** (**37%**)	3 (25%)	1 (14%)	4 (100%)	2 (17%)	7 (64%)
Internal validation						
Yes	**37** (**80%**)	12 (100%)	6 (85.7%)	0 (0%)	11 (92%)	8 (73%)
No	**9** (**20%**)	0 (0%)	1 (14.3%)	4 (100%)	1 (8%)	3 (27%)
Type of validation						
Cross	**20** (**43%**)	6 (50%)	4 (67%)	0 (0%)	3 (27%)	7 (78%)
Split	**12** (**26%**)	4 (33%)	0 (0%)	0 (0%)	7 (58%)	1 (11%)
Leave one out	**4** (**8.7%**)	2 (17%)	1 (17%)	0 (0%)	1 (9%)	0 (0%)
Bootstrap	**2** (**4.3%**)	0 (0%)	1 (17%)	0 (0%)	0 (0%)	1 (11%)
External validation						
Yes	**3** (**7%**)	1 (8.3%)	1 (14%)	0 (0%)	1 (8%)	0 (0%)
No	**43** (**93%**)	11 (91.7%)	6 (86%)	4 (100%)	11 (92%)	11 (100%)
Cohort						
OAI	**21** (**46%**)	3 (25%)	2 (29%)	1 (25%)	6 (50%)	9 (82%)
MOST	**5** (**11%**)	1 (8.3%)	0 (0%)	1 (25%)	2 (16.7%)	1 (9%)
CHECK	**3** (**7%**)	0 (0%)	2 (29%)	0 (0%)	0 (0%)	1 (9%)
Publicly available dataset						
Yes	**25** (**54%**)	3 (25%)	4 (57%)	3 (75%)	6 (50%)	9 (82%)
No	**21** (**46%**)	9 (75%)	3 (43%)	1 (25%)	6 (50%)	2 (18%)
Source code available						
Yes	**10** (**22%**)	2 (17%)	1 (14%)	0 (0%)	4 (33%)	3 (27%)
No	**36** (**78%**)	10 (83%)	6 (86%)	4 (100%)	8 (67%)	8 (73%)

CHECK, Cohort Hip and Cohort Knee; MOST, Multicentre Osteoarthritis Study; OA, osteoarthritis; OAI, osteoarthritis initiative; WND-CHARM, Weighted Neighbor Distance using Compound Hierarchy of Algorithms Representing Morphology.

### Number of patients

The number of patients in each study was heterogeneous. Indeed, the mean number of patients was 1359 and median 525 (range 18 –5749). Studies related to OA severity had a high number of patients, with mean 1803 and median 942 (range 18–4504); and studies related to phenotypes had the smallest number of patients, with mean 518 and median 559 (range 102–52). The first studies of ML in the field of OA were mainly related to diagnosis and OA prediction (median publication year 2017 (range 2008–2020)), whereas recent articles mainly focused on phenotype identification (median year 2018 (range 2015–2019)), and disease progression (median year 2019 (range 2012–2020)).

### Source of the data

Most of the analyses involved knee OA, 85% (N=39) of the articles, whereas only 15% (N=7) involved hip OA. No study investigated hand or foot OA. Overall, 64% of the articles described use of the OAI, the MOST and CHECK cohorts. The OAI database was described in 46% (N=21) of articles, the MOST database in 11% (N=5) and the CHECK database in 7% (N=3).

### ML methods

ML methods included the use of supervised algorithms, in 87% of articles (N=40), and unsupervised and semi-supervised algorithms, in 9% (N=4) and 4% (N=2). The most frequently used algorithms were convolutional neural network approaches. In total, 80% of the supervised algorithms were linked to classifications (N=32) and 20% (N=8) to regression analysis. Mixed ML algorithms were described in 28% (N=13) of articles. The ML methods differed according to the field of interest.

- In diagnosis articles, methods with convolutional neural network and random forest were predominant.
- Studies related to OA prediction involved methods such as elastic net regularisation and the multipurpose image classifier method: weighted neighbor distance using compound hierarchy of algorithms representing morphology (WND-CHARM).^73^
- Studies related to phenotypes involved unsupervised methods such as latent cluster analysis.
- Studies related to estimation of OA severity involved methods such as convolutional neural network and densely connected convolutional neural network.
- In progression-related articles, 45% (N=5) described methods derived from logistic regression.

DL algorithms were described in 35% of the selected articles (N=16) and in 75% (N=9) related to OA severity. Explainable ML was described in only 28% of the articles (N=13). Among the articles with unexplainable ML models, only 10/31 (32%) described interpretability tools, mainly gradient based. All studies related to phenotypes used explainable ML models, whereas all those related to OA severity used unexplainable ML models. A complete description of the algorithms used, and the interpretability tools is in online supplemental table S2.

### Type of data

The main type of data studied was imaging, in 74% (N=34) of articles; 61% (N=28) of the articles described analysis of X-ray data and 22% (N=10) MRI. Overall, 41% (N=19) of articles described analysis of clinical data, mainly demographic data and OA evaluation scores. Only 15% of the articles (N=7) described analysis of biological data in ML models, including 13% (N=6) patient serum and 4% (N=2) synovial fluid. Most of these studies focused on only one type of data, and only 24% (N=11) of articles described considering multiple data in their model (≥2 types of data), such as clinical and imaging data. Data analysis differed according to the field of interest:

- Articles related to OA prediction, representing 86% of articles, described use of imaging data (N=6), as compared with only 50% of articles related to OA early diagnosis.
- Studies related to OA severity estimation did not use biological data in their model.
- Studies related to phenotypes always used multiple types of data in their models, whereas those related to early diagnosis used a single type of data.

### Reproducibility

A separate training and testing set were described in 63% (N=29) of articles: internal validation in 80% (N=37), cross validation as the main internal validation method in 43% (N=20), leave one-out in 9% (N=4) and bootstrap in 4% (N=2). Only 26% (N=12) of the articles described splitting the cohort to validate the data. No validation was described in articles related to phenotypes, using unsupervised algorithms. External validation with an independent dataset was described only in 7% (N=3) of the articles. Datasets were described as publicly available in 54% (N=25) of the articles; however, source codes were available in only 22% (N=10).

## Discussion

**Figure 4:**
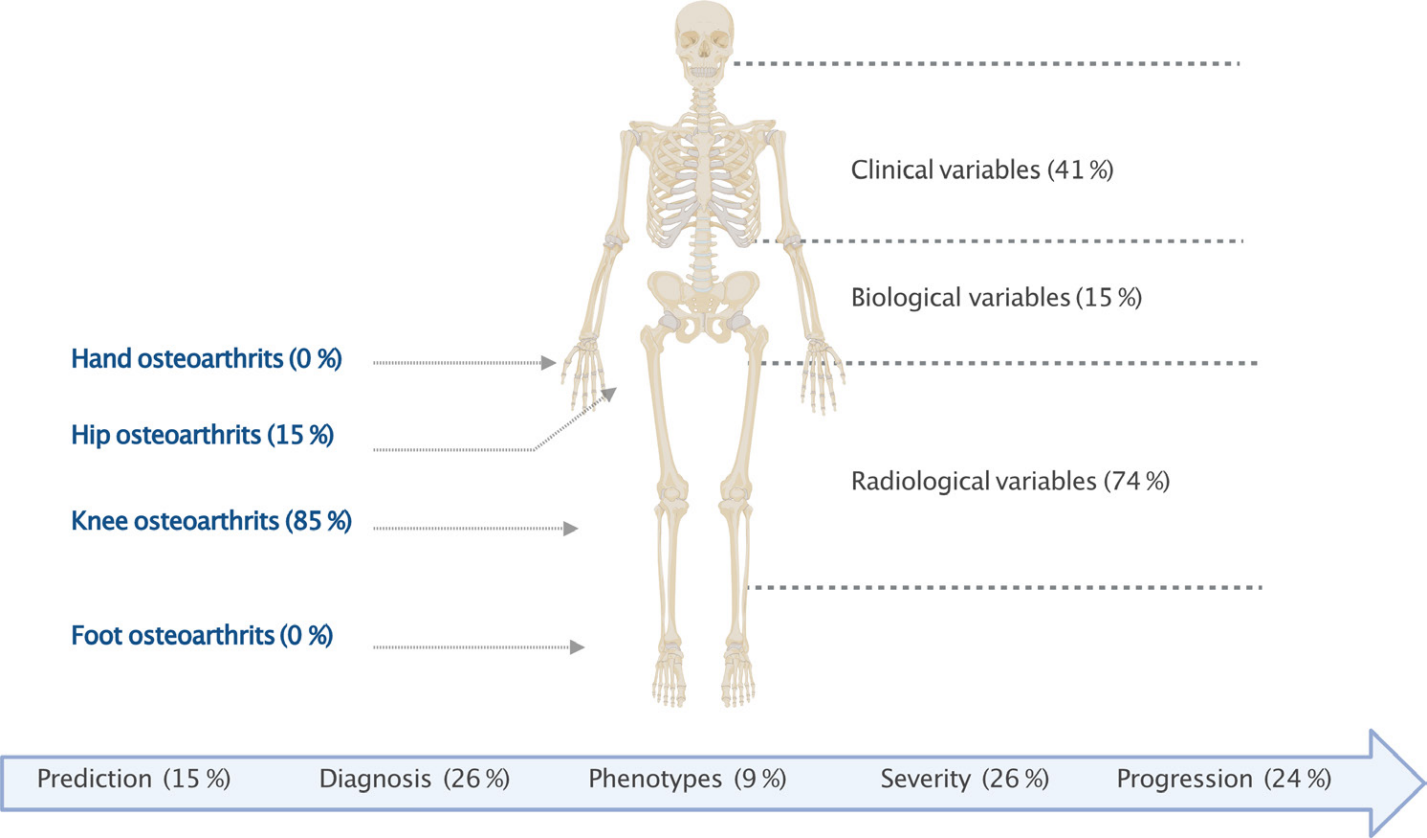
Overview of machine learning application in osteoarthritis.

This systematic review gives an exhaustive overview of ML approaches in OA research, currently a very dynamic field. Indeed, most of the articles related to ML in OA were published in the last 5 years. Our study highlights that (1) 85% of the ML articles focused on knee OA, mainly using the OAI database, with only 15% focusing on hip OA and none focusing on other sites such as hand or foot OA; (2) radiological data investigations predominated over clinical or biological data; (3) DL is increasingly being used and was described in 35% of the articles and (4) external validation was poorly used (7% of the articles) (figure 4).

Importantly, one major strength of applying ML in OA research is the wide range of clinical applications, covering the current scientific questions and main challenges in OA such as diagnosis of OA at an early stage, predicting the development of OA in the population, identifying OA phenotypes, estimating OA structural severity and identifying patients with slow and rapid disease progression. However, we found few articles on OA symptoms such as pain, function, or physical activity, and articles related to phenotypes were few (N=4), representing only 9% of the selected articles.

Our review revealed several limitations in how ML is applied in OA studies. First, most of the ML algorithms applied were based on supervised approaches, which may limit the power of identifying novel phenotypes based on data. In addition, we found high heterogeneity in terms of algorithms used depending on the study. Explainable ML was described in 33% (N=15) articles, and among the articles based on unexplainable ML models, only one third described interpretability tools (N=10/31 (32%)). These results highlight the need for increasing awareness of the need to develop explainable AI and ML models. Second, 74% of the selected articles were related to imaging, and few articles described the use of clinical and biological data, which limits the discovery of new phenotypes, biomarkers of severity progression or diagnosis. Third, the major focus on knee OA in studies using ML is questionable because of high heterogeneity among OA subtypes or localisations, which remains unclear and calls for diversifying the studies to better understand these diseases. With the several available hand-OA cohorts such as the Digital Cohort Design cohort,^74^ the Hand Osteoarthritis in Secondary care^75^ and the Nor-hand study,^76^ studies using ML tools in hand OA research are expected to better understand the characteristics, specificities and course of this OA localisation in the future. Finally, data and source codes for analyses were not available in 46% and 78% of the articles, but they are critical to ensure reliability and reproducibility of the ML analyses.^77 78^ Furthermore, external validation was described in only 7% of the selected articles, which is also a crucial point because ‘reproducibility crisis’ is one of the main challenges in science, particularly the ML field^14 79^

One major bias that could be highlighted in the studies applying ML to OA is that they mostly involved using the OAI, MOST and CHECK cohorts, with 46% of the articles involving the OAI database. Importantly, most of the cohorts currently available predate the interest of ML analyses in the field. Therefore, the study design and consent form may not have included broad data-sharing, which limits the use of the data for ancillary studies. This situation reinforces the need for purpose-built cohorts for ML analyses. As an example, the consortium for Applied Public–Private Research enabling OsteoArthritis Clinical Headway (APPROACH) aims at creating a broad OA multicentric cohort based on ML patient selection. APPROACH selected 297 patients with knee OA from five European established cohorts by using ML models. The patients will be further followed for 2 years, with additional data collection, and ML will be used to improve OA progression prediction.^80 81^

Finally, the definition of ML and AI are constantly evolving, so delineating articles using such approaches over time is difficult. In our review, we chose general terms and retrieved a large number of articles in our first selection, but rheumatology scientific societies should prompt for common language usage to ensure future reviews in the field.

To our knowledge, this is the first systematic review giving a comprehensive overview of the ML application in OA research. This work gathers the current applications of ML in OA and gives insights into several ways to enhance the ML application in research (summarised in box 1).

Box 1 **Prospective key points for osteoarthritis and machine learning research**

**Keys points**

- Increase the use of clinical and biological data.
- Use machine learning for other osteoarthritis sites (hand or foot).
- Establish additional cohorts.
- Improve reproducibility with external validation and data/source code availability.
- Develop machine learning checklists, consensus and training for the osteoarthritis scientific community.

We decided to focus on articles with direct clinical application in OA, so we excluded fundamental and theoretical articles in imaging, which are an important part of the current research in ML but did not fit our topic. Similarly, we excluded articles related to basic science and molecular biology, which are also increasingly using ML tools.^82^ We also excluded ML articles related to therapeutics because our study focused on the OA disease course and phenotypes. Articles related to OA surgery that were mainly based on robotic application and preoperative and postoperative prognosis were excluded. Because of high heterogeneity, we did not record the output of each article; however, we believe that these topics are of interest and the application of ML should also provide insights into the clinical care of OA patients.

Altogether, this work should prompt for more application of ML with analysis of clinical and biological data as well as symptoms of patients to discover new phenotypes, biomarkers of disease prediction, progression, and diagnosis. Our review results also strongly encourage the use of ML in hand OA because it is an important trait in OA, by taking advantage of available cohorts but also the development of additional ones. A better understanding of ML and its application is needed in our field and could be promoted by the development of specific ML consensus and training for the OA scientific community. The use of ML check-lists has been promoted in other fields^83 84^ and in an interventional clinical trial using ML according to the Consolidated Standards of Reporting Trials-Artificial Intelligence guidelines.^85^ The application of these checklists could improve the quality, standardisation, and reproducibility of ML studies in OA research.

In conclusion, ML is a fast-growing field providing better knowledge of human OA disease (diagnosis assistive tool especially for early OA, prediction for progression or severity of OA, characterisation of new therapeutic targets). This systematic review provides a comprehensive overview of ML applications in OA and delineates some methodological caveats that can and should be resolved to improve the quality of ML studies in OA research.

## Data Availability

All data relevant to the study are included in the article or uploaded as online supplemental information. All data relevant to the study are included in the article or uploaded as online supplemental information. Complementary data are available on request.
