# Supplementary material for: Use of machine learning in osteoarthritis research: a systematic literature review
Source: RMD Open. 2022 Mar 16;8(1):e001998. doi: 10.1136/rmdopen-2021-001998 (PMC8928401; doi:10.1136/rmdopen-2021-001998)
Supplement: Supplementary data [file rmdopen-2021-001998supp001.pdf]

Supplementary

Table S1 List of the 50 selected articles in Machine Learning and Osteoarthritis………2
Table S2 Table of data collected for each article

Binvignat M, *et al. RMD Open* 2022; 8:e001998. doi: 10.1136/rmdopen-2021-001998

Table S1 List of the 50 selected articles in Machine Learning and Osteoarthritis

diagnosis (N =12)

Title	**author**	**year**	**PMID**
Enabling early detection of osteoarthritis from presymptomatic cartilage texture maps via transport-based learning	Kundu S et al.	2020	32958644
Detection of hip osteoarthritis by using plain pelvic radiographs with deep learning methods	Üreten K et al.	2020	32248444
A Deep Neural Network-Based Method for Early Detection of Osteoarthritis Using Statistical Data	Lim J et al.	2019	30974803
A decision support tool for early detection of knee OsteoArthritis using X-ray imaging and machine learning: Data from the OsteoArthritis Initiative	Brahim A et al.	2019	30784984
Automatic Knee Osteoarthritis Diagnosis from Plain Radiographs: A Deep Learning-Based Approach	Tiulpin A et al.	2018	29379060
An evolutionary learning and network approach to identifying key metabolites for osteoarthritis	Hu T et al.	2018	29494586
Detecting knee osteoarthritis and its discriminating parameters using random forests	Kotti M et al.	2017	28242181
A preliminary examination of the diagnostic value of deep learning in hip osteoarthritis	Xue Y et al.	2017	28575070
Protein oxidation, nitration and glycation biomarkers for early-stage diagnosis of osteoarthritis of the knee and typing and progression of arthritic disease	Ahmed U et al.	2016	27788684
A computational method to differentiate normal individuals, osteoarthritis and rheumatoid arthritis patients using serum biomarkers	Heard BJ et al.	2014	24920114
Diagnosis of osteoarthritis and progression of tibial cartilage loss by quantification of tibia trabecular bone from MRI	Marques J et al.	2013	22941674
Identification of osteoarthritis biomarkers by proteomic analysis of synovial fluid	Han MY et al.	2012	23321181

prediction (N =7)

Title	**author**	**year**	**PMID**
An automated workflow based on hip shape improves personalized risk prediction for hip osteoarthritis in the CHECK study	Gielis WP et al.	2020	31604136
Bone texture analysis for prediction of incident radiographic hip osteoarthritis using machine learning: data from the Cohort Hip and Cohort Knee (CHECK) study	Hirvasniemi J et al.	2019	30825609

A machine learning approach for the identification of new biomarkers for knee osteoarthritis development in overweight and obese women	Lazzarini N et al.	2017	28899843
Predicting early symptomatic osteoarthritis in the human knee using machine learning classification of magnetic resonance images from the osteoarthritis initiative	Ashinsky BG et al.	2017	28084653
Simple Scoring System and Artificial Neural Network for Knee Osteoarthritis Risk Prediction: A Cross-Sectional Study	Yoo TK et al.	2016	26859664
Early detection of radiographic knee osteoarthritis using computer-aided analysis	Shamir L et al.	2009	19426848
Evaluation of a dynamic bayesian belief network to predict osteoarthritic knee pain using data from the osteoarthritis initiative	Watt EW et al.	2008	18999030

phenotype(N=4)

Title	**author**	**year**	**PMID**
Pain Susceptibility Phenotypes in Those Free of Knee Pain With or at Risk of Knee Osteoarthritis: The Multicenter Osteoarthritis Study	Carlesso LC et al.	2019	30307131
A machine learning approach to knee osteoarthritis phenotyping: data from the FNIH Biomarkers Consortium	Nelson AE et al.	2019	31002938
Using multidimensional topological data analysis to identify traits of hip osteoarthritis	Rossi-deVries J et al.	2018	29734501
Distinct subtypes of knee osteoarthritis: data from the Osteoarthritis Initiative	Waarsing JH et al.	2015	25882850

severity (N =11)

**Title**	**author**	**year**	**PMID**
Deep learning-based algorithm for assessment of knee osteoarthritis severity in radiographs matches performance of radiologists	Swiecicki A et al.	2021	33823398
A machine learning-based diagnostic model associated with knee osteoarthritis severity	Kwon SB et al.	2020	32978506
Toward automatic quantification of knee osteoarthritis severity using improved Faster R-CNN	Liu B et al.	2020	31938993
Semixup: In- and Out-of-Manifold Regularization for Deep Semi-Supervised Knee Osteoarthritis Severity Grading From Plain Radiographs	Nguyen HH et al.	2020	32804644

Binvignat M, et al. RMD Open 2022; 8:e001998. doi: 10.1136/rmdopen-2021-001998

Can a Convolutional Neural Network Classify Knee Osteoarthritis on Plain Radiographs as Accurately as Fellowship-Trained Knee Arthroplasty Surgeons?	Schwartz AJ et al.	2020	32418746
Development and Validation of a Multitask Deep Learning Model for Severity Grading of Hip Osteoarthritis Features on Radiographs	von Schacky CE et al.	2020	32013791
Predicting knee osteoarthritis severity: comparative modeling based on patient's data and plain X-ray images	Abedin J et al.	2019	30962509
Fully automatic knee osteoarthritis severity grading using deep neural networks with a novel ordinal loss	Chen P et al.	2019	31238184
Applying Densely Connected Convolutional Neural Networks for Staging Osteoarthritis Severity from Plain Radiographs	Norman B et al.	2019	30306418
3D convolutional neural networks for detection and severity staging of meniscus and PFJ cartilage morphological degenerative changes in osteoarthritis and anterior cruciate ligament subjects	Pedoia V et al.	2019	30306701
A fuzzy decision tree-based SVM classifier for assessing osteoarthritis severity using ground reaction force measurements	Moustakidis SP et al.	2010	20875766
Assessing hip osteoarthritis severity utilizing a probabilistic neural network based classification scheme	Boniatis I et al.	2007	16624611

progression (N=10)

Title	author	year	PMID
Serum adipokines/related inflammatory factors and ratios as predictors of infrapatellar fat pad volume in osteoarthritis: Applying comprehensive machine learning approaches	Bonakdari H et al.	2020	32561782
Prediction of Total Knee Replacement and Diagnosis of Osteoarthritis by Using Deep Learning on Knee Radiographs: Data from the Osteoarthritis Initiative	Leung K et al.	2020	32573386
Deep Learning Predicts Total Knee Replacement from Magnetic Resonance Images	Tolpadi AA et al.	2020	32286452
Multi-classifier prediction of Identification of osteoarthritis biomarkers by proteomic analysis of synovial fluidknee osteoarthritis progression from incomplete imbalanced longitudinal data	Widera P et al.	2020	32439879
Multimodal Machine Learning-based Knee Osteoarthritis Progression Prediction from Plain Radiographs and Clinical Data	Tiulpin A et al.	2019	31882803
Eight-year trajectories of changes in health-related quality of life in knee osteoarthritis: Data from the Osteoarthritis Initiative (OAI)	Törmälehto S et al.	2019	31323049

A Novel Method to Predict Knee Osteoarthritis Progression on MRI Using Machine Learning Methods	Du Y et al.	2018	29994316
MRI and biomechanics multidimensional data analysis reveals R(2) -R(1ρ) as an early predictor of cartilage lesion progression in knee osteoarthritis	Pedoia V et al.	2018	28471543
Development of a clinical prediction algorithm for knee osteoarthritis structural progression in a cohort study: value of adding measurement of subchondral bone density	LaValley MP et al.	2017	28511690
Osteoarthritis disease progression model using six year follow-up data from the osteoarthritis initiative	Passey C et al.	2015	25212288
A dissimilarity-based multiple classifier system for trabecular bone texture in detection and prediction of progression of knee osteoarthritis	Woloszynski T et al.	2012	23185959

Binvignat M, *et al. RMD Open* 2022; 8:e001998. doi: 10.1136/rmdopen-2021-001998

Table S2 Table of data collected for each article

group	names	PMID	number of patients	year	supervised/unsupervised ML used	mixed ML used	ML algorithms used	explainable ML used	interpretability tools used	DL used	type of OA studied	clinical data studied	biological data studied	serum data studied	synovium data studied	radiological data studied	X-ray data studied	MRI data studied	mutliple type of data co-analysed	training-testing data set available	model validation used	internal validation test type used	external validation test type used	cohort used	analysis script available	source data available
diagnostic	Tiulpin A et al.	29379060	3000	2018	supervised	No	Deep Siamese convolutional neural network	No	Gradient-weight activation map	Yes	knee	No	No	NA	NA	Yes	Yes	No	No	Yes	Yes	split dataframe	Yes	OAI | MOST	Yes	Yes
diagnostic	Hu T et al.	29494586	389	2018	semi-supervised	Yes	Evolutionary learning network	No	No	No	knee	No	Yes	Yes	No	No	NA	NA	No	No	Yes	cross validation	No	Else	No	No
diagnostic	Lim J et al.	30974803	5749	2019	supervised	No	Deep neural network	No	No	Yes	NA	Yes	No	NA	NA	No	NA	NA	No	Yes	Yes	split dataframe	No	Else	No	No
diagnostic	Brahim A et al.	30784984	1024	2019	supervised	Yes	Multivariate linear regression - Naives Bayes - Random Forest	Yes	NA	No	knee	No	No	NA	NA	Yes	Yes	No	No	No	Yes	leave one out	No	OAI	No	Yes
diagnostic	Kotti M et al.	28242181	94	2017	supervised	No	Random Forest	No	No	No	knee	Yes	No	NA	NA	No	NA	NA	No	Yes	Yes	cross validation	No	Else	No	No
diagnostic	Ahmed U et al.	27788684	225	2016	supervised	Yes	Random forests, multi- class logistic regression, multi-class sparse logistic regression, and support vector machines	No	No	No	knee	No	Yes	Yes	Yes	No	NA	NA	No	Yes	Yes	cross validation	No	Else	No	No
diagnostic	Han MY et al.	23321181	60	2012	supervised	No	Artifical Neural Network	No	No	No	knee	No	Yes	No	Yes	No	NA	NA	No	Yes	Yes	cross validation	No	Else	No	No
diagnostic	Marques J et al.	22941674	159	2013	supervised	No	Partial Least Square Linear Classifier	Yes	NA	No	knee	No	No	NA	NA	Yes	Yes	No	No	No	Yes	cross validation	No	Else	No	No
diagnostic	Xue Y et al.	28575070	210	2017	supervised	No	Convolutional Neural Network	No	No	Yes	knee	No	No	NA	NA	Yes	Yes	No	No	Yes	Yes	cross validation	No	Else	No	No
diagnostic	Heard BJ et al.	24920114	300	2014	supervised	No	Artifical Neural Network	No	Scale-conjugueted gradient	No	knee	No	Yes	Yes	No	No	NA	NA	No	Yes	Yes	split dataframe	No	Else	No	No
diagnostic	Üreten K et al.	32248444	434	2020	supervised	No	Convolutional Neural Network	No	No	Yes	hip	No	No	NA	NA	Yes	Yes	No	No	Yes	Yes	split dataframe	No	Else	No	No
diagnostic	Kundu S et al.	32958644	86	2020	supervised	Yes	Probabilistic Linear Discriminant Analysis	Yes	NA	No	knee	No	No	NA	NA	Yes	No	Yes	No	Yes	Yes	leave one out	No	OAI	Yes	Yes
phenotype	Nelson AE et al.	31002938	600	2019	supervised	No	Distant Weighted Discrimitation	Yes	NA	No	knee	Yes	Yes	Yes	No	Yes	Yes	No	Yes	No	No	NA	No	Else	No	Yes
phenotype	Waarsing JH et al.	25882850	518	2015	unsupervised	No	Latent Class Analysis	Yes	NA	No	knee	Yes	No	NA	NA	Yes	Yes	Yes	Yes	No	No	NA	No	OAI	No	Yes
phenotype	Carlesso LC et al.	30307131	852	2019	unsupervised	No	Latent Class Analysis	Yes	NA	No	knee	Yes	No	NA	NA	No	NA	NA	No	No	No	NA	No	MOST	No	Yes
phenotype	Rossi-deVries J et al.	29734501	102	2018	unsupervised	No	Topological Data Analysis	Yes	NA	No	hip	Yes	No	NA	NA	Yes	Yes	Yes	Yes	No	No	NA	No	Else	No	No
prediction	Shamir L et al.	19426848	129	2009	supervised	No	WND-CHRM	No	No	No	knee	No	No	NA	NA	Yes	Yes	No	No	Yes	No	NA	No	Else	Yes	No
prediction	Lazzarini N et al.	28899843	407	2017	supervised	No	RGIFE	No	No	No	knee	Yes	Yes	Yes	No	Yes	Yes	Yes	Yes	Yes	Yes	cross validation	No	Else	No	No
prediction	Ashinsky BG et al.	28084653	68	2017	supervised	No	WND-CHRM	No	No	No	knee	No	No	NA	NA	Yes	No	Yes	No	Yes	Yes	leave one out	No	OAI	No	Yes
prediction	Hirvasniemi J et al.	30825609	601	2019	supervised	No	Elastic net	Yes	NA	No	hip	No	No	NA	NA	Yes	Yes	No	No	Yes	Yes	cross validation	No	CHECK	No	Yes
prediction	Yoo TK et al.	26859664	1777	2016	supervised	No	Artifical Neural Network	No	gradient-descent learning algorithm	No	knee	Yes	No	NA	NA	No	NA	NA	No	Yes	Yes	cross validation	Yes	Else	No	No
prediction	Watt EW et al.	18999030	4796	2008	supervised	No	Bayesian Belief Network	Yes	NA	No	knee	Yes	No	NA	NA	Yes	Yes	No	Yes	Yes	Yes	cross validation	No	OAI	No	Yes
prediction	Gielis WP et al.	31604136	1002	2020	supervised	No	elastic net with automated parameter optimization	Yes	NA	No	hip	No	No	NA	NA	Yes	Yes	No	No	No	Yes	bootstrap validation	No	CHECK	No	Yes
prognosis	Törmälehto S et al.	31323049	3053	2019	supervised	No	Multinomial logistic regression, generalized estimating equation model	Yes	No	No	knee	Yes	No	NA	NA	No	NA	NA	No	No	No	NA	No	OAI	No	Yes
prognosis	Du Y et al.	29994316	100	2018	supervised	Yes	artificial neural network, support vector machine, random forest and naïve Bayes	No	No	No	knee	No	No	NA	NA	Yes	No	Yes	No	Yes	Yes	cross validation	No	OAI	No	Yes
prognosis	Pedoia V et al.	28471543	178	2018	unsupervised	No	Topological Data Analysis	Yes	NA	No	knee	No	No	NA	NA	Yes	No	Yes	No	No	No	NA	No	Else	No	No

6

prognosis	Woloszynski T et al.	23185959	137	2012	supervised	Yes	Support Vector Machine	No	No	No	knee	No	No	NA	NA	Yes	Yes	No	No	No	Yes	cross validation	No	Else	No	No
prognosis	Passey C et al.	25212288	1389	2015	supervised	No	Non linear mixed effect modeling	Yes	NA	No	knee | hip	Yes	No	NA	NA	No	NA	NA	No	No	No	bootstrap validation	No	OAI	No	Yes
prognosis	LaValley MP et al.	28511690	533	2017	supervised	No	Logistic regression	Yes	NA	No	knee	Yes	No	NA	NA	Yes	Yes	No	No	No	Yes	cross validation	No	OAI	No	Yes
prognosis	Leung K et al.	32573386	728	2020	supervised	No	Convolutional Neural Network	No	Gradient-weighted class activation mapping	Yes	knee	No	No	NA	NA	Yes	Yes	No	Yes	No	Yes	cross validation	No	OAI	Yes	Yes
prognosis	Tiulpin A et al.	31882803	2219	2019	supervised	Yes	Convolutional Neural Network	No	Gradient class activation map	Yes	knee	Yes	No	NA	NA	Yes	Yes	No	Yes	Yes	Yes	cross validation	No	OAI | MOST	Yes	Yes
prognosis	Widera P et al.	32439879	4467	2020	supervised	Yes	Random Forest	No	Shapley additive explanations methods	No	knee	Yes	No	NA	NA	Yes	Yes	No	Yes	No	Yes	cross validation	No	OAI | CHECK	No	Yes
prognosis	Tolpadi AA et al.	32286452	4796	2020	supervised	No	Convolutional Neural Network, random forest, support vector machine, logistic regresssion	No	No	Yes	knee	Yes	No	NA	NA	Yes	Yes	Yes	Yes	Yes	Yes	split dataframe	No	OAI	Yes	Yes
prognosis	Bonakdari H et al.	32561782	678	2020	supervised	Yes	Evolutionary algorithm particle swarm optimization, neuro-fuzzy inference system embedded with fuzzy c-means clustering	Yes	NA	No	knee	Yes	Yes	Yes	No	Yes	No	Yes	Yes	Yes	Yes	cross validation	No	OAI	No	Yes
severity	Pedoia V et al.	30306701	302	2019	supervised	No	Densely connected convolutional Neural Network	No	No	Yes	knee	No	No	NA	NA	Yes	No	Yes	No	Yes	Yes	split dataframe	No	Else	No	No
severity	von Schacky CE et al.	32013791	4368	2020	supervised	No	Convolutional Neural Network	No	Gradient-weighted class activation maps	Yes	hip	No	No	NA	NA	Yes	Yes	No	No	Yes	Yes	split dataframe	No	OAI	Yes	Yes
severity	Abedin J et al.	30962509	2951	2019	supervised	Yes	Elastic Net regression, weighted combination of LASSO and Ridge regression, convolutional neural network, random forest	No	NA	Yes	knee	Yes	No	NA	NA	Yes	Yes	No	Yes	No	Yes	split dataframe	No	OAI	No	Yes
severity	Norman B et al.	30306418	4504	2019	supervised	No	Densely connected convolutional Neural Network	No	No	Yes	knee	No	No	NA	NA	Yes	Yes	No	No	Yes	Yes	split dataframe	No	OAI	No	Yes
severity	Chen P et al.	31238184	4130	2019	supervised	No	Convolutional Neural Network	No	Gradient-weighted Class Activation Mapping	Yes	knee	No	No	NA	NA	Yes	Yes	No	No	Yes	Yes	split dataframe	No	OAI	Yes	Yes
severity	Boniatis I et al.	16624611	18	2007	supervised	No	Probabilistic Neural Nettwork	No	No	No	hip	No	No	NA	NA	Yes	Yes	No	No	Yes	Yes	leave one out	No	Else	No	No
severity	Liu B et al.	31938993	1385	2020	supervised	No	region proposal network Convolutional neural network	No	Stochastic **Gradient** Descent	Yes	knee	No	No	NA	NA	Yes	Yes	No	No	Yes	Yes	cross validation	Yes	Else	No	No
severity	Moustakidis SP et al.	20875766	36	2010	supervised	Yes	Support Vector Machine	No	No	No	knee	Yes	No	NA	NA	No	NA	NA	No	Yes	Yes	cross validation	No	Else	No	No
severity	Kwon SB et al.	32978506	357	2020	supervised	Yes	Random Forest	No	No	No	knee	Yes	No	NA	NA	No	NA	NA	No	No	Yes	hold-out validation	No	Else	Yes	No
severity	Nguyen HH et al.	32804644	500	2020	semi-supervised	Yes	Semix-up	No	stochastic gradient descent,	Yes	knee	No	No	NA	NA	Yes	Yes	No	No	Yes	Yes	cross validation	No	OAI | MOST	Yes	Yes
severity	Schwartz AJ et al.	32418746	288	2020	supervised	No	Convolutional Neural Network	No	No	Yes	knee	No	No	NA	NA	Yes	Yes	No	No	Yes	No	NA	No	Else	No	No
severity	Swiecicki A et al.	33823398	2802	2021	supervised	No	Region Proposal Network	No	No	Yes	knee	No	No	NA	NA	Yes	Yes	No	No	Yes	Yes	split dataframe	No	OAI | MOST	No	Yes
